# Gender-Specific Differences in Outcome of Ascending Aortic Aneurysm Surgery

**DOI:** 10.1371/journal.pone.0124461

**Published:** 2015-04-22

**Authors:** Carsten J. Beller, Mina Farag, Sepali Wannaku, Philipp Seppelt, Rawa Arif, Arjang Ruhparwar, Matthias Karck, Alexander Weymann, Klaus Kallenbach

**Affiliations:** Heart Centre Heidelberg, Clinic for Cardiac Surgery, University of Heidelberg, Heidelberg, Germany; University of Crete, GREECE

## Abstract

**Objectives:**

Gender specific differences receive increasing attention and are known to affect the outcome of cardiovascular diseases. We investigated possible risk-factors for gender-specific differences in ascending aortic aneurysm surgery.

**Methods:**

548 consecutive patients (male: n = 390, age: 58.3±14.4 years; female: n = 158, age: 65.3±12.9 years) with aneurysms of the ascending aorta eligible for cardiac surgery were retrospectively analyzed.

**Results:**

Women were significantly older when operation was indicated (p<0.001) and presented with significantly more hypertension (p=0.04) and chronic obstructive pulmonary disease (COPD; p = 0.017), whereas men had significantly more previous cardiac operations (p = 0.016). Normalized aortic diameters (diameter / body surface area) were significantly larger in women (3.10±0.6 cm) vs. (2.75±0,5 cm, p≤0.001) in men, without differences in absolute values (5.74±1.04 cm vs. 5.86±1.34 cm). The aortic arch was significantly more involved in aneurysm formation in women (p = 0.04). Follow-up was available in 93% of the patients with a mean follow-up time of 3.9±3.9 (0-17.8) years. 30-day mortality was 3.5% in men (n=12) and 7.9% in women (n=11; p = 0.058). Univariate regression analysis shows gender specific risk factors for 30-day mortality in men to be age: p = 0.028; myocardial infarction: p = 0.0.24 and in women diameter of the ascending aorta: p=0.014; renal insufficiency: p=0.007. Long-term survival was significantly reduced in women (log-rank p = 0.0052).

**Conclusions:**

The outcome after surgery for ascending aortic aneurysm is less favourable in women with significantly reduced long-term survival and a trend to increased 30-day mortality in this cohort. Larger normalized aortic diameters, higher incidence of involvement of the aortic arch and differences in comorbidities may contribute to gender differences. Women undergo surgery at higher age and more progressed state of aortic disease. Therefore, gender-specific guidelines for ascending replacement may be useful to improve outcome in women.

## Introduction

In recent years, there is increasing attention for gender-specific differences regarding prevalence, treatment and outcome of cardiovascular disease. For coronary artery disease and recently for mitral valve surgery as well for heart-transplantation gender-specific differences in outcome are described [1–3]. There are also well-documented gender differences in abdominal aortic aneurysm disease and outcome after surgical treatment [4–6]. So far only few studies have focused on possible gender differences in thoracic aortic disease.

Risk-factors for thoracic aortic aneurysms include besides genetic syndromes such as Marfan syndrome and bicuspid aortic valves, hypertension, smoking and chronic obstructive pulmonary disease. Recently, our group demonstrated that four different established surgical techniques for treatment of ascending aortic aneurysms resulted in low mortality and low reoperation rate and prevented aortic dissection [7]. Furthermore, we observed gender-specific differences in long-term outcome. Therefore, in the present study, we investigated this patient cohort in particular for possible gender-specific risk-factors and differences in outcome of ascending aortic aneurysm surgery.

## Patients and Methods

### Patients

Retrospective review of our institutional database revealed 548 patients, who were operated on for aneurysm of the ascending aorta from January 1994 till September 2011. Patients with acute aortic dissection, size reduction aortoplasty and those with sole replacement of the non-coronary sinus in addition to ascending aorta replacement were excluded. Data collection was done by chart review. Follow-up was obtained after written consent and approval of institutional review board (Name: Ethics Committee University of Heidelberg; S-286/2010) through contact with the local population administration office, family doctor or the patient/family directly (only adult patients who are legally competent were included). Follow-up was complete in 93% of the cases."

### Surgical procedure and selection of surgical strategy

Choice of surgical procedure (supracommissural aortic replacement (SCR), composite replacement (CR) and aortic valve-sparing using David´s or Yacoub´s technique) and surgical strategy were described previously in detail [7]. Briefly, indication for ascending aortic replacement was given at a diameter of ≥5.5 cm and more recently (since October 2006) at 5cm. Arch involvement was defined by an aortic diameter greater than 40mm. However arch involvement did not lead to routine arch replacement, since the current surgical cutt-off value is much higher. Different surgeons performed operations for SCR, CR and David´s technique, whereas one surgeon only from 1997 till 2003 applied the Yacoub technique. SCR was applied to patients with normal aortic root or to older patients with only slightly enlarged aortic root or who were not robust enough for a complex root surgery. The aortic valve was replaced either with a biological or mechanical prosthesis, if necessary. David’s technique was first applied in October 2006 and since then used as the only valve-sparing technique, if applicable.

Median sternotomy was the standard surgical approach in all cases; CPB was established by direct aortic and right atrial or bi-caval cannulation cooling to a rectal temperature of 32–34°C. For arch surgery deep (18–22°C) and more recently moderate hypothermia (26–28°C) with additional cold ante-grade cerebral perfusion by direct intubation of the supra-aortic branches was used. Myocardial protection was performed using repetitive doses of cold crystalloid or blood cardioplegia in an ante-grade fashion after aortic cross clamping.

### Statistical analysis

Categorical data are shown as percentage, and continuous variables are shown as mean ± standard deviation as well as median and range. The study group was split according to sex into two different study arms and preoperative, operative and postoperative data were evaluated.

If Gaussian distribution was given, Student’s t test was used for continuous variables; otherwise Mann-Whitney U Test was performed. Categorical variables were analyzed by Fisher’s Exact—Test. Univariate logistic regression was accomplished in both study arms to define relevant risk factors for 30-day mortality. Risk factors for gender-specific late mortality were analyzed with Cox regression. Multivariate analysis for early and late mortality was omitted due to limited sample size. Kaplan-Meier analysis was used to estimate late mortality. A probability value less than 0.05 (two-tailed) was considered significant. Statistical analysis was performed by SPSS 21.0 software ((SPSS, Chicago, IL).

## Results

### Patient characteristics

**Fig 1:**
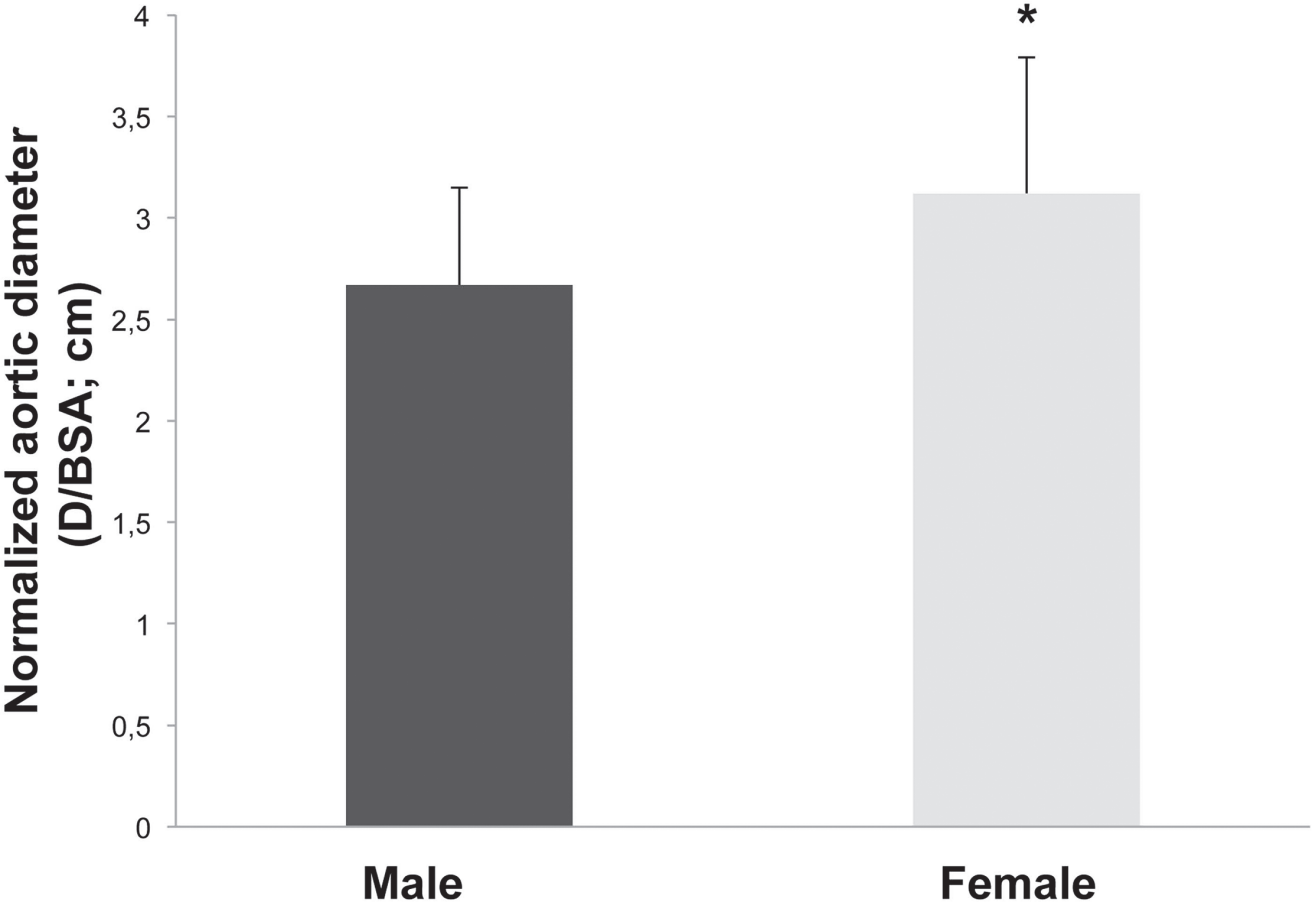
Normalized aortic diameter, calculated as absolute diameter/body surface area. * = difference is significant.

Regardless of the gender, indication for surgery was given at 5.5 cm or, more recently, at 5.0 cm dilatation of the ascending aorta. Several perioperative characteristics differed between male and female patients and are demonstrated in Table 1. Male patients had significantly more previous cardiac operations, whereas female patients were significantly older, showed more aortic arch involvement, suffered more often from hypertension (treated or untreated) and presented more often with COPD. Furthermore, women had significantly larger normalized ascending aortic diameters (ratio of diameter to body surface area) with 3.1 ±0.6 cm versus 2.75 ± 0.5 cm in men (p = 0.016) (Fig 1), albeit the absolute aortic diameters showed no significant difference between genders (5.74 ± 1.04 cm in women vs. 5.86 ± 1.34 cm in men; p = n.s.). Other risk factors like NYHA class, diabetes mellitus or renal insufficiency were distributed evenly between both genders.

**Table 1:** Summary of patient’s characteristics.

Variable	Male n = 390 (71)	Female n = 158 (29)	P-value
Age, year	58.3 ± 14.4	65.3 ± 12.9	<0.001
BMI, kg/m^2^	27.18 ± 4,71	26.06 ± 5.22	0.099
Ascending aortic diameter, mm	58.6 ± 13.4	57.4 ± 10.4	0.139
Normalized ascending aortic diameter, mm (diameter/BSA)	27.5 ± 5	31.0 ± 6	0.016
Arch involvement	15 (4.3)	13 (9.6)	0.04
Arch and descending aortic involvement	8 (2.3)	5 (3.7)	0.4
Thoracoabdominal aortic involvement	10 (2.9)	3 (2.2)	0.9
Marfan´s syndrome	19 (5.4)	10 (7.4)	0.5
Bicuspid aortic valve	41 (25.8)	13 (20.0)	0.4
Emergency operation	15 (4)	9 (5.9)	0.3
Previous cardiac operations	26 (16.6)	3 (4)	0.016
Recent myocardial infarction	35 (9.1)	9 (5.7)	0.3
Endocarditis	3 (0.9)	1 (0.7)	0.89
Hypertension	282 (72.7)	131 (83.4)	0.04
-*Treated*	163 (42)	68 (43.3)	
-*Untreated*	119 (30.7)	63 (40.1)	
Diabetes	38 (9.8)	14 (8.9)	0.1
-*Dietary treatment*	11 (2.8)	3 (1.9)	
-*Oral treatment*	17 (4.4)	10 (6.4)	
-*Insulin treatment*	10 (2.6)	1 (0.6)	
NYHA class			0.1
*-I*	71 (18.5)	20 (12.7)	
*-II*	135 (35.2)	45 (28.7)	
*-III*	129 (33.6)	71 (45.2)	
*-IV*	49 (12.5)	20 (13.4)	
Renal insufficiency	27 (14)	13 (15.3)	0.9
Creatinine, mg/dl	1.01± 0.3	0.86± 0.26	<0.001
COPD	35 (9)	34 (21,3)	0.018

BMI, body-mass index; BSA, body surface area; NYHA, New York Heart Association; COPD, chronic obstructive pulmonary disease, arch involvement: diameter ≥ 40mm, renal insufficiency when creatinie concentration ≥ 1.5mg/dl

### Perioperative and early outcome

Operative values are shown in Table 2. Operation duration, CPB time, cross clamp time or the need of concomitant surgical intervention did not differ significantly between male and female patients. Since women showed more aortic arch involvement, the relative need for circulatory arrest was higher compared to men: 27% (n = 43) vs. 17.7% (n = 67; p = n.s.). However circulatory arrest duration was similar in both groups (19.6 ± 18.1 min for men vs. 19.6 ± 14.0 min for women).

**Table 2:** Summary of operative data.

Variable	Male n = 390 (71)	Female n = 158 (29)	P-value
Operation time, min	273.3 ± 94.8	271.4 ± 85.5	0.8
Bypass time, min	159.5 ± 65.8	155.6 ± 58.2	0.5
Cross-clamp time, min	102.2 ± 36.9	101.6 ± 39.9	0.8
Circulatory arrest, min	19.6 ± 18.1	19.6 ± 14.0	0.45
Hemiarch replacement	22 (33.3)	17 (39.5)	
Complete arch-replacement	5 (7.6)	3 (7)	
Aortic-arch and descending replacement	8 (12.1)	5 (11.5)	
Open aortic inspection and anastomosis	32 (48.5)	18 (41.9)	
Additional procedures			
*-CABG*	52 (15.1)	15 (10.9)	0.3
*-AVR*	41 (10.5)	20 (12.6)	1.0
*-MVR*	9 (2.6)	3 (2.2)	1.0
Surgical technique			0.004
*-Composite replacement*	(57.2)	75 (47.5)	
*-Supracommissural aortic replacement*	104 (26.7)	50 (31.6)	
*-Aortic valve-sparing*, *David technique*	59 (15.1)	24 (15.2)	
*-Aortic valve-sparing*, *Yacoub technique*	4 (1)	9 (5.7)	

CABG, coronary artery bypass grafting; AVR, aortic valve replacement; MVR, mitral valve replacement

Perioperative values are shown in Table 3. The stay on ICU was significantly longer for women compared to men (4.4 ± 6.7 d vs. 3.1 ± 3.7 d; p = 0.024), who also needed more dialysis treatment (5.8% vs. 1.2%; p = 0.024).

**Table 3:** Perioperative results.

Variable	Male n = 390 (71)	Female n = 158 (29)	P-value
Invasive ventilation time, h	56.9±150.5	48.2±132.6	0.5
Chest tube bleeding, ml	1524.8±2420	1367.3±1714	0.5
Re-thoracotomy	20 (5.3)	13 (8.4)	0.17
Low cardiac output			0.33
*-Conservative treatment*	7 (1.8)	5 (3.2)	
*-IABP*	7 (1.8)	1 (0.6)	
*-ECC*	1 (0.3)	0 (0.0)	0
Neurological complications			
*-TIA*	0 (0)	1 (0.7)	0.28
*-Stroke*	2 (0.6)	4 (2.9)	0.059
Dialysis	4 (1.2)	7 (5.8)	0.016
Stay on ICU, day	3.1±3.7	4.4±6.7	0.024
Hospitalization, day	11.9±8	11.9±12	0.95
Thirty-day mortality	12 (3.5)	11 (7.9)	0.058
Late mortality	19 (5.6)	13 (9.4)	0.78
Overall mortality	31(9.1)	24 (17.3)	0.017
Mean follow-up, year	3.99±4	3.75±3.6	0.5
Cause of death			
*-Cardiac*	4 (1)	5 (3)	0.456
*-Non-cardiac*	14 (3.5)	13 (8.2)	0.13
*-Aortic*	4 (1)	1 (0.6)	0.387
*-Neurological*	4 (1)	3 (1.9)	1.0
Reoperation			
*-Ascending aortic reoperation*	5 (1.6)	1 (0.6)	1.0
*-Aortic valve reoperation*	4 (1)	1 (0.6)	1.0
*-Ascending aortic and aortic valve reoperation*	5 (1.6)	1 (0.6)	1.0
*Pacemaker implantation*	15(5)	5(4.2)	0.8

IABP, intra-aortic balloon pump; ECC, extracorporeal circulation; TIA, transient ischemic attack; ICU, intensive care unit

Thirty-day mortality was 7.9% for women vs. 3.5% for men, but did not reach statistical significance (p = 0.058). Other variables including neurological complications, need for re-thoracotomy, ventilation time and overall hospital stay did not show significant differences between groups.

### Long-term follow-up

Mean follow-up time did not differ between groups (3.99 ± 4 yrs. for men vs. 3.75 ± 3.6 yrs. for women; p = n.s.) with a span of 0 to 17.6 years. Late mortality was nearly double in the women’s group compared to men (9.4% vs. 5.6%, p = n.s.); however, the overall mortality of 17.4% in the women’s group was significantly higher compared to 9.1% in the men’s group (p = 0.017) as shown in Table 3. Actuarial survival was significantly higher in men when compared to women with 92±1.7 vs. 84±3.6% at 5 years and 80±4.5% vs. 70±8% at 10 years (log-rank p = 0.0052) [as shown in 7]. During that period, need for re-operation was evenly distributed between the two groups.

### Risk factors for 30-day mortality and long term survival

Using univariate regression analysis, several parameters could be identified as risk factors influencing 30-day mortality as shown in Table 4. Concerning men, age (HR = 1.146; p = 0.028), previous myocardial infarction (HR = 8.33; p = 0.024) and preoperative need for Coumadin therapy (HR = 1.562; p = 0.002) proved to be statistically significant predictors.

**Table 4:** Univariate regression: risk factor analysis for early (30-day) mortality.

**Men**	**P-value**	**HR (95% CI)**
Age	0.028	1.146 (1.015–1,295)
Myocardial infarction	0.024	8.333 (1.328–52.307)
Previous Coumadin therapy	0.002	1.562 (1.173–2.079)
**Women**	**P-value**	**HR (95% CI)**
Diameter of ascending aorta	0.14	1.631 (1.102–2.414)
Arch involvement	0.009	14.267 (0.928–105.560)
Arch and descending aortic involvement	0.027	27.750 (1.459–527.982)
Thoracoabdominal aortic involvement	0.048	13.750 (1.022–185.037)
Urea	0.028	1.062 (1.006–1.121)
Creatinine	0.013	23.082 (1.933–275.681)
Renal insufficiency	0.007	9.333 (1.844–47.237)

HR, hazard ratio; CI, confidence interval, arch involvement: diameter ≥ 40mm, renal insufficiency when creatinie concentration ≥ 1.5mg/dl

For women, 7 risk factors were found to be significant for 30-day mortality: diameter of the ascending aorta (HR = 1.6; p = 0.014), aortic arch involvement (HR = 14.3; p = 0.009), aortic arch and descending aorta involvement (HR = 27.8; p = 0.027), thoracoabdominal aortic involvement (HR = 13.8; p = 0.048), preoperative urea concentration (HR = 1.1; p = 0.028), preoperative creatinine concentration (HR = 23.1; p = 0.013) and renal insufficiency (HR = 9.3; p = 0.007).

Analysis of gender-specific risk factors for late mortality by univariate cox regression is summarized in Table 5. Concerning men, several variables were identified, with urgency (HR = 4.38, p = <0.001), previous CABG operation (HR = 2.56, p = 0.026), post operative central nervous system complications (HR = 3.56, p = 0.012) and need for dialysis treatment postoperatively (HR = 14.11, p = 0.001) having the greatest influence on survival. Other variables were age, previous myocardial infarction, preoperative urea concentration, cardiopulmonary bypass and circulatory arrest time. Preoperative aortic valve insufficiency had a positive influence on survival (HR = 0.44, p = 0.042).

**Table 5:** Univariate Cox Regression: Gender-specific risk factor analysis for late mortality.

**Men**	**P-value**	**HR (95% CI)**
Age	0.00	1.071 (1.033–1.100)
Urgency	0.00	4.383 (1.922–9.995)
Aortic valve insufficiency	0.042	0.443 (0.202–0.970)
Myocardial infarcion	0.000	1.457 (1.180–1.800)
Urea	0.049	1.019 (1.000–1.039)
Previous cardiac surg.	0.026	2.563 (1.116–5.886)
Bypass time	0.000	1.006 (1.003–1.009)
Circulatroy arrest	0.018	1.027 (1.005–1.050)
CNS complications	0.012	3.562 (1.322–9.595)
Dialysis treatment	0.001	14.109 (3.073–64.779)
**Women**	**P-value**	**HR (95% CI)**
Diameter of ascending aorta	0.000	1.536 (1.224–1.927)
Circulatory shock	0.013	4.971 (1.395–17.714)
Smoking	0.002	2.058 (1.305–3.246)
NYHA I	0.043	0.082 (0.007–0.922)
NYHA II	0.021	0.068 (0.007–0.666)
Urea	0.003	1.042 (1.014–1.071)
Creatinine	0.000	20.529 (4.208–100.1529)
Renal insufficiency	0.000	7.421 (2.838–19.400)
Bypass time	0.005	1.008 (1.002–1.014)
Circulatory arrest	0.041	1.036 (1.001–1.072)

HR, hazard ratio; CI, confidence interval; CABG, coronary artery bypass grafting; CNS, central nervous system; NYHA, New York Heart Association, renal insufficiency when creatinie concentration ≥ 1.5mg/dl

For women NYHA I state and NYHA II had a positive influence on survival (HR = 0.082, p = 0.043 and HR = 0.068, p = 0.021 respectively), reflecting relatively better clinical state at the time of operation. Variables with the greatest impact on mortality were preoperative creatinine concentration (HR = 20.53 p = <0.001), and renal insufficiency (HR = 7.42, p = <0.001) as well as preoperative circulatory shock (HR = 4.97, p = 0.013). Other predictors of late mortality were: the diameter of the ascending aorta, history of smoking, preoperative urea, as well as cardiopulmonary bypass time and circulatory arrest time. Time in hospital, and time spent on ICU were also significant surrogate parameters reflecting patients with higher comorbidities. Detailed depiction of HR, 95% CI and P-values are found in Table 5.

## Discussion

It is already accepted since the introduction of the EuroScore that female gender and age are independent risk factors for coronary artery bypass surgery. Recently, different studies showed females to have less favorable results considering late mortality after aortic root surgery [8–9]. This encouraged our group to search for gender-specific risk factors influencing early and late mortality and possibly providing an explanation for this observation. To the best of our knowledge, this is the first study trying to elucidate gender- specific risk factors.

Normalized aortic diameters (diameter/body surface area) were significantly larger in women than in men, without differences of absolute values. The aortic arch was significantly more involved in aneurysm formation in women, whereas no gender differences were observed for other diseased parts of the aorta.

There was a trend to increased 30-day mortality in women. In female gender, the absolute aortic diameter, aortic arch, arch and descending, or arch and thoracoabdominal aortic involvement were negative predictors for 30-day mortality. Further significant negative predictors for early mortality in women were preoperative urea and creatinine concentrations and renal insufficiency. Significantly lower 30-day survival in women after surgical treatment of ascending aortic aneurysms was reported from another study [10]. After adjustment for age, cardiopulmonary bypass time, re-thoracotomy and postoperative elevated creatinine the early mortality risk for women was even higher [10]. In male gender, age, previous MI and Coumadin therapy were identified as risk factors for early mortality, probably reflecting the different comorbidities of men in our study. So far—to our knowledge—no specific predictors for early mortality in men have been analyzed by other studies.

In concurrence with the aforementioned studies [8, 9], our population showed similar differences in late mortality, with a significant survival advantage for male gender considering late mortality. Analysis of preoperative characteristics showed age and previous MI as significant risk factors for late mortality in men. Furthermore, the urgency of the procedure was unveiled as a negative predictor of mortality in men. In women however, circulatory shock was established to be a specific risk factor of late mortality. Other preoperative risk factors exclusive to women were history of smoking, the aortic diameter, urea and creatinine concentrations and the presence of renal insufficiency.

Not surprisingly, CPB and circulatory arrest time were intraoperative variables negatively influencing late survival in both men and women.

Postoperative occurrence of CNS complications and need for dialysis treatment were negative predictors of outcome for male gender. Whereas in women the time spent on ICU, or at the hospital until discharge, were negative postoperative predictors of outcome, probably as surrogate parameters reflecting more complicated postoperative course. Therefore, we did not weigh theses parameters as independent risk factors

The only significant positive predictor of survival in the men’s group was the preoperative presence of AR, while the presence of NYHA classes I or II in women had also a positive impact on survival. Both findings probably reflect a better clinical state at the time of operation.

Prior work has clarified the cumulative, life-time risk of rupture or dissection based on the size of thoracic aneurysms and patients were mostly divided according to the presence or absence of connective tissue disorders, the morphology of the aortic valve, age group and comorbidities. Hypertension, smoking and COPD are known independent risk factors, but few studies have examined postoperative outcome with regard to gender [11–12].

It is well known that aneurysm size has a profound impact on rupture, dissection, and death. Patients presenting with ascending aortic aneurysms, exceeding 5.5 cm in diameter are advised to undergo protective aortic-replacement surgery, with or without the aortic root, the aortic arch, and the descending aorta, according to the underlying pathology, to prevent rupture or dissection. The guidelines were often revised through outcome analysis and risk factor stratification, a trend has emerged to reduce the cut off value for surgery to 5 cm, and 4.5 cm for specific risk groups, such as patients with Marfan Syndrome or other genetic connective tissue disorders [13–14] So far, indexing of aortic diameter for BSA is recommended only for patients of small body size (BSA ≤1.68 m^2^) [15]. In specific patient groups like Turner Syndrome, it has already been shown that a normalized aortic diameter greater than 2.0 cm/m^2^ already presents an aortic enlargement and diameters greater than 2.5 cm/m^2^ predispose for aortic dissection [16]. Hence, the current general guidelines for ascending aortic replacement, which do not account for indexed values, may lead to further progression of the disease in women with subsequent aortic arch involvement and significantly larger normalized aortic diameters as seen in our study.

In our study, women were significantly older and did in fact show significantly more COPD, hypertension, and more arch involvement, which are well-known risk factors associated with worse clinical outcome, whereas other risk factors such as smoking history, diabetes or preoperative renal insufficiency were similarly distributed in both genders. On the other hand, the positive predictive impact of NYHA class I and II in females reflects the fact that these patients showed less comorbidities and thus had a favorable outcome. Similarly the presence of preoperative AR in men was associated with a positive outcome, which may be related to the fact that these patients were probably candidates for AVS techniques such as the David procedure, a group of patients usually showing low comorbidities.

In summary, female patients have worse outcome and higher mortality rates when affected with aortic disease (AAA, TAA or Type A dissection). They tend to be older when afflicted with these diseases [5, 17–18], or according to our findings present a more advanced clinical stage of aortic disease and are probably therefore older, until they meet current criteria for surgery. Implementation of these new gender-specific findings in the next revision of the guidelines for ascending aortic aneurysms may be helpful to improve outcome in women.

Possible explanation for the delayed presentation and older age might be the estrogen-mediated reduction of MMP-2 and MMP-9 synthesis [19]. However failure in medical recognition and management of the disease in women might also play an important role, which leads to the need of additional gender-specific approaches to the diagnosis [11]. In a recent study, women with aortic disease were found to be significantly older than men and represented less than 25% of patients less than 40 years [19]. The absolute diameters and lengths of the aorta were greater in men when compared to women, however after adjusting for BSA women showed greater diameter/BSA ratio as in our study. This suggests that the smaller diameters of the aorta progresses rapidly in female sex with advanced age since the absolute diameters were near equal in the 7^th^ decade [20]. Hence not only the diameter of the aorta reflects the severity of the disease, but the rate of progression seems to play as well an important role in the natural history of aortic disease.

In conclusion, this study strongly supports a gender-specific assessment of thoracic aortic aneurysms, including the creation of gender-specific guidelines for prophylactic ascending aortic replacement.
